# Content Generalizability: Generalizing Qualitative Research to the General Study Population

**DOI:** 10.3390/mps9030090

**Published:** 2026-06-04

**Authors:** Costas S. Constantinou

**Affiliations:** Department of Basic and Clinical Sciences, University of Nicosia Medical School, Nicosia 2417, Cyprus; constantinou.c@unic.ac.cy

**Keywords:** content generalizability, generalization, qualitative research, qualitative inquiry, data saturation

## Abstract

This paper highlights an important topic in qualitative research: the generalizability of qualitative findings to the general study population. While the concept of generalizability in qualitative inquiry has been discussed, with types such as analytical generalizability and transferability being proposed, the direct generalization of qualitative results to the general population has received limited attention. This oversight stems largely from the prevailing belief among researchers that qualitative research cannot be generalized due to its reliance on small, non-randomized samples. This perception has led many scientists, particularly those outside the social sciences, to regard qualitative research primarily as exploratory or supplementary to quantitative findings. This paper is a technical note that presents a new concept and procedure. Based on concept analysis, *content generalizability* is proposed, which offers a new type of generalizability through which qualitative findings can be applied to the general population, thereby enabling them to inform policy and drive social change. *Content generalizability* could expand the uses of qualitative research within the scientific community by providing knowledge and insights into the experiences of the general study population. This paper also contributes to developing empirical research by testing and applying *content generalizability*.

## 1. Introduction

### 1.1. Reflection

Generalizability in qualitative research has been discussed and documented, resulting in controversies, debates, and uncertainties [1,2,3,4]. Before delving deeper into the relevant literature, I would like to reflect on two cases of PhD examinations that exclusively relied on qualitative inquiry and show the problem of generalizability of qualitative research findings.

The first one relates to the completion of my PhD a few years ago. I recall one of the examiners expressing curiosity about how my ethnographic study on the experience of end-stage kidney disease could inform policy. At the time, I felt confident enough to respond promptly, stating that my study could only provide insights specific to the context, as it was not generalizable to the broader population of patients with end-stage kidney disease. The examiner seemed satisfied with my answer, which reinforced what I had already understood. That is, the purpose of qualitative research is to deeply explore specific groups within particular contexts, rather than to generalize findings to a wider population, echoing Lincoln and Guba’s early argument that qualitative research cannot be generalized [5]. At the time, I felt certain and safe, but, at the same time, I thought how more useful my study would be if I could generalize my findings to inform policy and change.

The second PhD examination was fifteen years later with one of my PhD students. She had conducted a qualitative study examining how nurses perceived and experienced autonomy in the workplace, employing focus groups and rigorously coding data to construct themes. Approximately forty nurses participated in her study. One of the examiners, with more experience in quantitative research, asked whether the perspectives of these forty participants were sufficient to draw reliable conclusions and inform policy. I expected her to respond as I had fifteen years earlier, explaining that her findings were specific to the participants and could not be generalized to all nurses in the country. Instead, to my surprise—and concern—she replied, “yes”. I worried that this response might suggest ignorance of the widely accepted principle that qualitative research was not intended for generalization. However, she elaborated, explaining that she had many years of experience in nursing and nursing education, and that she had talked with many more nurses outside her study. She advised that she did not hear anything different from what her participants expressed during the focus group discussions. While her answer might have seemed subjective, it was contextually grounded. She spoke confidently, emphasizing that she had achieved data or thematic saturation and had gathered sufficient depth to answer her research questions effectively [6,7]. Interestingly, her response introduced the possibility of generalizing qualitative findings to a broader population within the study’s scope, along the lines of what many other qualitative researchers had claimed [3].

The two cases above are not only indicative of the controversies around the generalizable nature of qualitative research but also of the uncertainties and sometimes anxieties that qualitative researchers might experience every time they have to justify the usefulness of their study. Such controversies and uncertainties are also captured in the literature as discussed below.

### 1.2. Background and Justification

In the literature, qualitative research has been criticized as less useful than quantitative research because its results could not be generalized [8,9]. The issue of generalizability of qualitative research was discussed initially in the 1980s during the ‘paradigm wars’ between qualitative and quantitative researchers [10]. That was the time when Lincoln and Guba [5] (p. 110) explained that qualitative research did not aim at any generalizations, making the popular statement ‘The Only Generalization Is: There is No Generalization’, and suggesting ‘transferability’ (explained later in the paper) as the most suitable alternative, which was later accepted as the most commonly used term or the gold standard of generalizability in qualitative inquiry. Lincoln and Guba were so influential that qualitative researchers widely accepted that qualitative research could not and should not be generalized. The end-result was that generalizability of qualitative research was highly ignored, with many textbooks not even mentioning it [10].

As a way to compensate for this lack of generalizability in qualitative research and respond to the criticism that qualitative research was not as important and valuable for sciences as quantitative research was, new types of generalizabilities were introduced such as analytical generalizability, transferability [2], situated, internal and external generalizability, and so forth [4]. All these types of generalizability are presented below in further detail as part of the concept analysis. Despite the ignorance of generalizability of qualitative research to broader populations, many qualitative researchers have presented their results as generalizable and usable for policy making and intervention [3], causing further controversy and uncertainty around the generalizable power of qualitative research.

Aiming to provide more clarity and certainly, more recently, Roald et al. [11] published an article entitled ‘Why do we always generalize in qualitative research?’. Roald et al. questioned the distinction between ‘idiographic’ sciences (the humanities and social sciences) and ‘nomothetic’ sciences (natural sciences), challenging the long-standing criticism that qualitative research cannot be generalized. The authors emphasized that, in practice, all research, qualitative or quantitative, provides findings from specific observations to broader conclusions or understandings. Roald et al. noted that generalizations in qualitative research are less obvious than in quantitative research because qualitative research explores context-rich cases which are not reduced to measurable variables. They carried on explaining that the criticism or claim that qualitative research cannot be generalized because it explores specific cases is not correct. In fact, specific cases very often give substantial knowledge, as they reveal more general trends in the population. They used examples such as Sigmund Freud’s and Charles Darwin’s to indicate that individual cases in both natural and social sciences were linked with broader theoretical tenets. Furthermore, they explained that findings from phenomenological studies develop insights that go beyond individual cases. Conclusively, Roald et al. maintained that it is not a matter whether qualitative research can be generalized but a matter of which generalization is best to make based on the aims of each study, elaborating more on naturalistic and analytical generalizations.

Importantly, in response to Roald et al.’s article, Osbeck and Antczak [4] wrote that qualitative researchers themselves rejected the idea of generalizability of qualitative research because of its link with positivism and objectivity, whereas qualitative research emphasized the uniqueness of contextualized human experience and the need to give voice to vulnerable and marginalized groups. They invited other scholars to comment on Roald et al.’s approach, aiming to expand the discussion and knowledge around the concept of generalizability. Some advised that qualitative researchers should provide clarity in the generalizability power of their study and clarify in which way their study could be generalized. Another scholar encouraged qualitative researchers to ensure that the typical and diverse experiences were representative of the studied cases, or to provide all necessary information that can be used or applied by other researchers in other contexts. Interestingly, none of these scholars endorsed or expanded on Roald et al.’s idea that qualitative research can draw broader conclusions by deriving knowledge from individual cases. Such reluctance to take Roald et al.’s approach further might have been reflected in the long-standing assumption that qualitative research results cannot be generalized to broader populations. This assumption or claim is so strong that many qualitative researchers include lack of ability to generalize their qualitative findings as a limitation of their study [1,12].

The main contribution of Roald et al.’s approach was that it was maybe the first time that qualitative research claimed strongly it was generalizable to broader cases, even though no clear procedure was provided regarding how this could be achieved. Moreover, none of the invited researchers in Osbeck and Antczak’s paper suggested any new types of generalizability that would break the dominant belief that qualitative research could not be generalized to the broader population. On this note, this paper is a concept analysis of generalizing qualitative research results to the general study population, and it provides new conceptualization and an overall procedure of generalizing qualitative research findings to the general study population.

## 2. Materials and Methods

The method used in this short study is based on Walker and Avant’s [13] ‘concept analysis’ framework. Concept analysis aims to explore how a concept is used, clarifying its uses and proposing new dimensions or a case model. It has eight steps, namely (1) select a concept, (2) determine the aims or purposes of analysis, (3) determine the defining attributes, (4) identify all uses of the concept, (5) identify a model case, (6) identify borderline, related, contrary, etc., (7) identify antecedents and consequences, and (8) define empirical referents.

Below, the results are presented as per the concept analysis steps. Since this is a technical note paper, which is not expected to be a lengthy article as per the journal’s guidelines, it aimed neither to conduct a systematic review of the literature or analysis nor to engage in deep conceptual analysis of generalizability. Instead, it discusses how the term is used in the basic literature and the controversies around it. Therefore, Scopus and PubMed were the main data sources, while Google Scholar stood as supplementary. The keywords used were ‘generalization’, ‘generalizability’, ‘qualitative research’, ‘qualitative inquiry’, and ‘study population’. Having said this, only articles that presented or discussed specific types of generalizabilities in qualitative research were consulted. Inclusion criteria were as follows: papers that exclusively discussed types of generalizability in qualitative research, papers that discussed how qualitative findings can be generalized to broader populations, empirical studies, concept or theoretical papers, no specific publication dates to capture any early theoretical papers. Papers that did not include exclusively qualitative studies, discussed mixed methods, and were not published in English were excluded. The review of the papers was undertaken by one reviewer who is the author of this paper.

Searching was a challenging endeavor because the initial search yielded several thousands of articles. A more targeted search, such as ‘generalization and qualitative research’, showed 2527 results, which were screened by their title. This screening indicated 87 papers that seemed relevant to the inclusion criteria and were therefore screened from their abstract and full-text reading in cases that were not clear. This task resulted in 23 papers that were included in this concept analysis (reference numbers: 1–5; 8–14; 16–26). Although no paper discussed generalizing qualitative research to broader populations as a distinct type, papers that discussed other types of generalizability in qualitative research were considered.

## 3. Results

### 3.1. Selected Concept

This is to clarify that this concept analysis paper does not analyze the concept of generalizability in qualitative research. This has been performed by other scholars such as Smith [12] or Osbeck and Antczak [4]. Instead, based on the reflective part in the Introduction of this paper and controversies around generalizability in qualitative research, the concept selected is ‘generalizing qualitative research results to the study or broader population and how it is achieved’.

### 3.2. Aim of the Analysis

The aim of the analysis is to understand how the concept is used, if used at all, and what procedure it encompasses and construct or generate new dimensions or a new concept and procedure.

### 3.3. Defining Attributes

The defining attributes include the following: (1) Generalization of qualitative research results to the general study population is a type of generalizability in qualitative inquiry. Such generalizability does not necessarily refer to statistical generalization but how representative the results are of the general population under study. (2) A procedure for this type of generalizability. (3) To achieve this type of generalizability, qualitative results are required to be enough to ensure adequate representation in the study population.

### 3.4. Uses of the Concept

The purpose here is not to exhaust the literature or perform a systematic literature review but to explore the basic references to the selected concept. The relevant literature that outlines specific types of generalizability in qualitative inquiry clearly establishes that qualitative research cannot be generalized to the broader population in the same way as quantitative research [1]. To elaborate, in quantitative survey-based social research for example, sampling is determined based on calculations of statistical power, the size of the population under study, and random selection of participants—widely regarded as the gold standard for ensuring generalizability [8]. The data collected in such quantitative studies consists of numbers and scores, which are later analyzed using statistical software. This approach is fundamentally inapplicable to qualitative research, which typically involves small samples and focuses on collecting and analyzing words rather than numerical data [9,14,15]. This means that the selected concept of this study is not used as a distinct type of generalizability in qualitative inquiry.

Despite its incompatibility with statistical–probabilistic generalization, qualitative research has its own forms of generalizability, as discussed in the literature [2,16]. Below, the focus is on four more recognizable types, namely naturalistic generalizability, analytical generalizability, transferability, and designed generalization, while reflecting on their limitations, as well as on a few more types which have more recently been developed in response to discussing the concept of generalizability.

‘Naturalistic generalizability’ refers to the process in which readers or scholars recognize parallels between research findings and their own experiences or knowledge [17]. If the findings resonate with their personal or professional experiences, the research achieves naturalistic generalizability. Conversely, if the findings do not align, it does not imply that the research lacks utility; rather, it may present opportunities to explore alternative perspectives [18]. Achieving this type of generalization requires researchers to provide rich, detailed descriptions that allow readers to determine whether the findings align with their experiences. However, a notable limitation is that naturalistic generalizability depends entirely on the reader’s interpretation. Researchers themselves are not involved in this process and often cannot ascertain whether their study achieves generalization. As a result, this form of generalization may appear to have limited impact on scholarship.

Another type of generalizability is ‘analytical generalizability’, which involves extending findings to broader concepts or theories [14,19,20]. This can occur through demonstrating how results align with or challenge established ideas, developing new theories, or reinterpreting existing ones using different methodologies. Analytical generalization is particularly valuable for advancing scholarship, as it contributes to theoretical development, refines existing concepts, and enriches the literature on specific topics or issues. However, it does not address the relevance of findings to the general population or their capacity to inform policy or drive social change. Its contributions are largely limited to providing theoretical insights.

A third type of generalizability of qualitative research is ‘transferability’, which occurs when readers or researchers identify potential applications of findings in their own contexts [1,5,20]. In essence, the findings of one study can be used in another context to make comparisons or explain discrepancies. Drisko [21] clarified that transferability is a form of generalization, but the transfer of knowledge produced in one study to other contexts is not performed automatically, like what is happening in the traditional form of generalization, but it is critically determined by the users of the qualitative study. While transferability is similar to analytical generalizability in fostering academic or theoretical advancement, it also has limited influence on policy or social change. Like analytical generalization, transferability is subjective, relying on the discretion of other researchers to determine the relevance of findings from one study to another. This reliance on subjective criteria often results in little concrete evidence of generalizability.

Falk and Guenther [22] proposed an alternative approach to generalizing qualitative research, which they called Designed Generalization from Qualitative Research (DGQR). They argued that qualitative research can be generalized and have broader implications if it is designed with that purpose in mind. They introduced a generalization cycle that begins with ‘normative truth statements’ and evolves through the refinement of these statements via research. This process involves engaging stakeholders and policymakers to effectively communicate the applicability of the research findings. While Falk and Guenther’s proposed method of designed generalization offers a valuable and interesting approach, it appears to be more suited to case-based studies. Additionally, this form of generalization does not aim to apply findings to broader populations but rather focuses on context-specific issues.

In addition to the above-mentioned types of generalizability, other scholars suggested other ways of generalizing results in qualitative inquiry. For example, ‘moderatum generalization’ means that qualitative results are generalized in the sense that any broad insights are subject to change being transformed into long-standing conclusions [9]. Maxwell [23] distinguished between ‘internal’ and ‘external’. Internal generalization refers to ensuring that the results are representative of the typical views and experiences as well as any diverse approaches expressed by the participants. External generalization is on par with transferability and means that the results might be applicable in other contexts. Schraube and Højholt [24] suggested ‘situated generalization’ to explain generalization ‘through subjectivity-in-context’ in the sense that subjective experiences or individual cases are part of the wider socio-cultural and historical practices. Interestingly, Levitt [25] talked about ‘qualitative generalization’, which refers to developing or identifying the variations in the data that represent the experience of the phenomenon; hence, qualitative research is generalized to the phenomenon but not to the population. Levitt’s approach concept seems closer to ‘analytical generalization’. Mayring [26] has also talked about making generalization from case studies, but the generalization was not to be made to broader populations but to other similar contexts on par with transferability, provided that multiple cases are considered, and in longitudinal analysis.

Conclusively, the generalizable power of qualitative research to the broader population has not been used as a distinct type. It seems that the types described above have been an attempt to establish that qualitative research cannot be generalized like quantitative research, guided by Lincoln and Guba’s initial argument, but it is still useful because it can be generalized in other ways.

### 3.5. Model Case

From the outline of the uses of the selected concept above, it does not seem there is an available model of generalizing qualitative research results to the general study population. Roald et al. [11] touched on this possibility but did not provide deeper conceptualization or a practical procedure. On this note, I have constructed a case model which comprises a new type of generalizability and has all defining features. In Section 3.8, the procedure whereby *content generalizability* can be achieved is presented.

The idea for generalizing qualitative data to a broader population is reflected in the fact that qualitative research has nothing to generalize but words. These words are not predetermined but are generated through the research itself and mapped afterward with a coding process. Comparing qualitative research against survey-based quantitative inquiry helps better understand why generalizing words is plausible in qualitative research, prompting the exploration of alternative methods to make qualitative data applicable to broader populations. Specifically, in quantitative social research, for example, participants select answers from an existing questionnaire. In this context, the range of possible responses is predetermined, and participants simply choose the option that best represents them. Thus, these possible answers are what could be selected by the entire population. With randomization and large samples, quantitative researchers want to ensure that the distribution of answers is representative of the broader population. Consequently, it becomes essential to identify an appropriate sample before data collection.

In qualitative research, however, the range of possible responses is not predetermined. Instead, it is developed afterward through an extensive process involving open-ended questions and systematic coding. As a result, qualitative researchers produce the landscape of possible responses and are not interested in how these responses are distributed in the population but in achieving depth. While quantitative research restricts participants to selecting from predefined options—often failing to adequately capture the complexity of their experiences and thoughts due to the rigidity of numbered scales—qualitative research allows for greater depth, which is achieved gradually conducting more research if necessary to generate more words or content. Put differently, qualitative research enables researchers to delve into the nuances of human behavior, capturing complexities that can potentially represent a broader population beyond the study participants.

Building on the ability of qualitative research to delve deeply into participants’ experiences, thoughts, and perspectives, generating a detailed landscape, the concept of *content generalizability* is proposed here as a means to generalize qualitative findings and make them applicable to broader populations.

*Content generalizability* refers to the ability to generalize the organized content derived from qualitative data to the general population under study. In simpler terms, the organized or thematic content—in this paper, it is called ‘thematic landscape’—could represent what the broader study population might say about the issue under investigation. For example, consider the research of the PhD student mentioned in the Introduction who studied nurses and perceived autonomy. If all members of the study population (i.e., nurses in the country where the PhD research was conducted) were interviewed or participated in focus groups, their responses would likely be categorized into the same codes, themes, and subthemes constructed through the coding of data from the sampled participants. This is made possible through the combination of both the depth and breadth of the sampled participants’ views and experiences. Qualitative research often goes so deeply into participants’ perspectives that all possible viewpoints or experiences are encompassed in broader categories, such as themes. While some individuals in the broader population might emphasize certain aspects over others, their responses would still fall within the spectrum of themes or codes identified from the sampled participants. Thus, what is generalized in *content generalizability* is not statistical probability or the representativeness of a sample, as in quantitative research, but the thematic content of the sampled participants’ views, experiences, or narratives. Unlike Onwuegbuzie and Leech’s (2010) [3] argument that qualitative research requires random sampling for generalization, random selection is neither relevant nor necessary in this context. The aim is not probabilistic or statistical generalization but generalization of content. While quantitative generalizability asks ‘how is this distributed across the population?’, *content generalizability* asks ‘what are the things this population could say about this phenomenon?’. Therefore, the primary criterion for generalizability in qualitative research is to ensure sufficient depth and breadth of responses.

To conclude the concept of *content generalizability*, reflecting on a hypothetical case is useful. It should be noted here that the following example is only hypothetical and, like *content generalizability* itself, should be tested empirically. Suppose we conduct twenty interviews to understand how patients with AIDS experience stigma in x country, ensuring both breadth of experiences and depth of exploration until data saturation is reached. After the completion of the study and the coding and analysis of the results, a question arises: what additional insights would emerge if we interviewed every patient with AIDS in the country or a representative number of them randomly selected? Within the framework of *content generalizability*, it is reasonable to hypothesize that no new insights would fall outside the spectrum of themes or the thematic landscape already identified from the twenty interviews. Here, it is essential to unpack ‘thematic landscape’. ‘Thematic’ does not merely refer to the broad themes identified in the final stage of the coding process. Instead, it includes all results of the coding process, namely codes, subthemes, themes, and the overarching findings under each theme. All these are structured meanings and interpretations of how people make sense of the phenomenon. Therefore, this is like a landscape where all these separate components of the coding process are hosted and communicate with each other to support the same purpose. It is the content of this thematic landscape that is generalizable and is representative of the general study population. In other words, what *content generalizability* ultimately asserts is that the structured landscape of meaning, experience, and perception that interpretivist analysis produces is not merely a portrait of the few people studied. It is a knowledge map of what the phenomenon looks like across the population. With ‘qualitative generalization’, Levitt [25] argued that qualitative research can be generalized to the phenomenon, not to the population. With ‘content generalization’, an appropriate rephrase is ‘qualitative research can be generalized to the population not only to the phenomenon’.

### 3.6. Borderline, Related, and Contrary Cases

Borderline, related, or contrary cases refer to instances where some attributes of the concept are used or the concept is misused. It is not feasible to outline all such cases separately, but Onwuegbuzie and Leech’s [3] work has been illuminating. They explored 125 qualitative articles published in the journal Qualitative Report looking for instances of generalizing the results to the wider population. They considered that 29.6% made inappropriate generalizations based on qualitative results. They cited specific studies to support their finding. For instance, they argued that D’Cruz’s study of caregivers for HIV/AIDS patients improperly suggested that its findings could inform interventions. Similarly, they critiqued Turner, whose study made broader conclusions that women with no or grown children had stronger relationships with companion animals than women with younger children. According to Onwuegbuzie and Leech, these studies crossed a line by drawing quantitative-like conclusions and attempting statistical generalization. However, both studies contained rich data, and their authors did not claim to be making statistical or numerical generalizations. Rather, they sought to draw broader conclusions that extended beyond their immediate findings. It seems that their attempt was not essentially different from what the PhD student in the Introduction of this paper suggested with her response to the examiner, or what Roald et al. described regarding the generalizable power of qualitative research.

This criticism seems rooted in the assumption that interpretivist inquiry, characterized by small sample sizes and in-depth exploration, cannot adequately represent the broader population. While it is true that small sample sizes cannot achieve statistical or numerical representation, as already discussed, there is no evidence to suggest that rich, detailed qualitative data cannot representatively reflect the broader population’s perspectives.

### 3.7. Antecedents and Consequences

Antecedents are situations or experiences that preceded and influenced the development of the concept. In this case, what preceded the development of *content generalizability* was the experience with the PhD student who had to respond to an examiner who had quantitative research and statistical generalizability in their mind. Additionally, the concept of ‘naturalistic generalizability’, which relates to a match between qualitative findings and personal experiences, placed the grounds for searching for a type of generalizability that would validate this felt match. Finally, Roald et al.’s [11] approach about generating broader conclusions from individual cases contributed to formulating a clearer direction.

Consequences refer to what happens after the occurrence of a phenomenon. *Content generalizability* is a newly proposed type, but the insights from Onwuegbuzie and Leech’s [3] discussion showed that the qualitative researchers who used the term ‘generalized results’ were criticized or were considered as misleading readers. Importantly, *content generalizability* provides a framework and a safety umbrella that qualitative researchers can use to further substantiate their study.

### 3.8. Empirical Referents

Empirical referents aim to identify how the concept should be used, implemented, and measured. Achieving generalizability of qualitative data to the broader study population requires the following basic steps: (1) Clear research questions that align with the study’s aims. (2) Well-designed questions for the participants that can generate deep content. (3) Rigorous coding and thematic analysis to ensure accurate categorization of data. A ‘thematic network’ could be generated along the lines of Attride-Stirling’s [27] framework or a detailed coding landscape or the thematic analysis framework by Braun and Clarke [28,29], which includes a detailed mapping process of identifying keywords, codes, and themes. These should be accompanied by the overarching findings under each theme in order to build a sufficient thematic landscape. (4) Data or thematic saturation, validated through appropriate methods. This is important to ensure that qualitative researchers have enough data to answer their research questions and build a rich thematic landscape. Also, having enough data is reflected in other basic quality markers in qualitative research, which should also be ensured and articulated during reporting of the study. In addition to transferability already outlined in this paper, credibility, dependability, and confirmability are important markers to consider [5]. Credibility means that the research findings represent participants’ experiences. Dependability refers to the documentation of the research process and that it is reproducible. Confirmability establishes that the findings derive from participants’ responses and do not reflect the researchers’ subjective understanding. (5) Adhere to reporting standards for qualitative research [30,31].

By adhering to these principles above, researchers can ensure the depth necessary for *content generalizability*, allowing the codes, themes and subthemes derived from qualitative data to represent the broader population under study. *Content generalizability* is more applicable to research in which data or thematic saturation is relevant and achievable, such as the General Inductive Approach and thematic analysis [32], Ethnographies [33], and even Interpretive Phenomenological studies [34,35]. It could also be considered for qualitative content analysis or textual analysis.

Additionally, qualitative researchers could conduct studies to empirically test the concept. More specifically, they could undertake a two-stages study. That is, in the first stage, conduct qualitative interviews along the lines of the five steps outlined above and on the basis of purposive or convenient sampling to generate a thematic landscape. In the second stage, they could select a representative sample and randomly select participants to see how their responses or experiences are placed in the thematic landscape generated from the previous stage. This two-stage approach is on par with a combination of inductive (first stage) and deductive coding (second stage) analysis.

## 4. Discussion

The PhD student’s response to her examiner, as described in the Introduction of this paper, demonstrated ‘naturalistic generalization’, as her findings resonated with her own observations. At the same time, she opened the door to the possibility of generalizing her results to the broader population of nurses. Her extensive experience and observations led her to believe that involving all nurses in focus groups or interviews would not yield insights significantly different from those derived from the forty participants in her study. This confidence stemmed from the depth of her exploration of nurses’ experiences with professional autonomy. She believed that any additional perspectives or experiences from other nurses would still fit within the spectrum of themes already identified. Possibly the qualitative researchers in Onwuegbuzie and Leech’s [3] study experienced similar feelings, hence they presented their findings as generalizable. Through concept analysis, *content generalizability* is a proposed type of generalizability of qualitative inquiry and aims to provide more certainty to qualitative researchers about the generalization of their results and the use for informing policy and interventions.

Additionally, *content generalizability* could transform how qualitative research is currently perceived and utilized, particularly in fields outside the social sciences, such as medicine and applied sciences, where qualitative research is largely underestimated or considered subpar. For instance, Gagliardi and Dobrow [36] found that qualitative research articles accounted for only 0% to 0.6% of publications in general medical journals and 0% to 6.4% in health services and policy research journals. Sidhu, Jones, and Stevenson [37] (p. 229) underscored the importance of qualitative research in medicine but also highlighted challenges in publishing such research in medical journals. They noted a ‘[…] sharp relief in a recent exchange of correspondence in the BMJ that highlighted a clear policy to exclude qualitative research due to the view that results are largely exploratory’.

Reflecting this perception, Pyo et al. [38], more recently, in an article published in the Journal of Preventive Medicine & Public Health, outlined five cases in which qualitative research was applicable. These are as follows: (1) exploratory when a topic is not well known; (2) to better understand quantitative findings; (3) to explain something that cannot be explained adequately with existing knowledge; (4) to explain the reasoning for theoretical development; (5) when detailed descriptions are required. However, Pyo et al. [38] did not present qualitative research as an independent or standalone method capable of adequately investigating phenomena, let alone as a basis for policymaking or social change. Instead, their description implied that qualitative research served primarily as an exploratory or supplementary tool for quantitative inquiry. 

*Content generalizability* as a concept and procedure has a few limitations, which can be addressed. First, there is no research evidence to support *content generalizability*. Before empirical testing conceptualization is required; hence this article. The way *content generalizability* has been developed seems to reflect the approach by other scholars who initially conceptualized types of generalizability in qualitative research. Qualitative researchers could test *content generalizability* empirically by engaging deeply with reflexivity to make sure that any preexisting biases either in favor or against do not intervene and by employing a thorough process of quality assurance (e.g., two independent coders to validate the thematic landscape).

Second, to achieve *content generalizability*, qualitative researchers should rely heavily on data saturation, a concept that has been contested. It is true that data saturation has been debated and that many qualitative researchers do not consider it useful [39,40]. At the same time, there are other scholars who have endorsed and used it in their studies (examples found in [6,41,42]), while it has been incorporated in the COREQ (Consolidated Criteria for Reporting Qualitative Research) [31] and the SRQR (Standards for Reporting Qualitative Research) [30] guidelines for reporting qualitative research. The criticism is valid, as saturation very often appears as a technical procedure which seems against the philosophical and epistemological grounds of qualitative research. However, what needs to be further explored and discussed is that qualitative research as scientific inquiry is expected to formulate research objectives or questions. Therefore, without data saturation, how do qualitative researchers know they have enough data to answer these questions? Or how do they decide the number of their participants knowing that this number is enough for interpretivist analysis? In other words, data saturation should not be approached as substituting interpretivist analysis but only as a way to decide whether further such analysis is needed.

Third, even with data saturation achieved, it is not guaranteed that qualitative researchers would identify all possible codes and themes. This can indeed potentially happen. However, if qualitative researchers strictly follow a quality assurance process with at least two independent coders, employ a saturation method, and present a thematic network or a detailed code/thematic mapping along the lines of the thematic analysis guidelines, this challenge should be overcome.

Fourth, *content generalizability* does not seem like it can capture the more special or unique cases. However, these special cases would be like the outliers or the statistical error in quantitative research. This is not to equate these special cases with outliers or statistical error, but to clarify that in qualitative research we may have key or dominant findings and results different from these key findings. In any case, the dominant thematic landscape could still be generalizable.

Fifth, some could argue that *content generalizability* is not based on a theory but largely derived from experience. In fact, it is reflected in the interpretive thought and analysis because it does not offer a substitute to any other types of generalizability or to the foundational purpose of qualitative research, i.e., that of in-depth understanding of contextualized human experience. What is generalized with *content generalizability* is itself interpretivist knowledge. That is, structured meanings and interpretations of how people live, feel, and make sense of the world, derived from interpretivist analysis. *Content generalizability* adds more importance to qualitative studies by generalizing their confirmed thematic landscape.

Sixth, another counterargument could be that *content generalizability* is not essentially different from any study that employs the thematic framework of analysis and achieves saturation. Certainly, *content generalizability* has not been born from parthenogenesis. Its innovation lies with the fact that it illuminates what was deeply buried or hidden in the argument and belief that qualitative research cannot be generalized to broader populations. In addition, some could argue that focusing too much on generalizing qualitative research to the population could potentially undermine interpretivist epistemology or at least what Onwuegbuzie and Leech called ‘interpretive consistency’ [3]. This could happen largely if qualitative research aimed for statistical generalization; *content generalizability* suggests generalizing the thematic landscape of experiences’ content which, arguably, enhances the importance interpretivist epistemology in the sense that the underlined structure, or thematic landscape, of interpretivist analysis represents the broader population under study.

As explained above, despite any possible limitations, *content generalizability* has the potential to elevate qualitative research to a new level within the scientific community, where it is regarded as equally important and necessary as quantitative research, especially by disciplines outside the social sciences and humanities. By advancing beyond its traditional roles—such as exploring specific groups, conducting exploratory studies, developing theories, offering insights, and complementing quantitative findings—qualitative research can provide insights into the experiences and perspectives of entire populations, informing policy and driving social change.

## 5. Conclusions

It is notable that qualitative researchers rarely discuss the possibility of generalizing qualitative data to the broader population. This may be because such generalization is often associated with statistical and probabilistic approaches, which are fundamentally incompatible with the philosophy and practice of qualitative inquiry. Through concept analysis, the concept of *content generalizability* is introduced to generalize qualitative findings to the general population without compromising the essence of qualitative research or interpretivist approaches.

*Content generalizability* suggests that the thematic landscape derived from data collected through qualitative methods—organized into codes, subthemes, themes, and overarching findings and achieved through saturation—represents the perspectives of the broader population under study. In essence, the views, experiences, or narratives of sampled participants are reflective of what could be expected from the entire population within the scope of the research. Adopting and following a specific procedure, *content generalizability* could significantly enhance the influence of qualitative research by positioning it as a more impactful tool for policymaking and social change. It could open new research directions in qualitative inquiry and extend its relevance to disciplines beyond the socio-cultural and humanistic fields.

## Data Availability

The original contributions presented in this study are included in the article. Further inquiries can be directed to the corresponding author.
